# Critical Medical Ethics as an Approach to the Debate About Assisted Suicide by the Example of Germany

**DOI:** 10.1111/bioe.70061

**Published:** 2025-11-28

**Authors:** Meike Gerber

**Affiliations:** ^1^ Institute for the History of Medicine, Faculty of Medicine, Dresden University of Technology Dresden Germany

**Keywords:** assisted suicide, autonomy, critical theory, euthanasia, principlism

## Abstract

Recent literature has seen a growing endorsement of the so‐called autonomy‐only approach to assisted dying, which rejects suffering as a necessary criterion for access. Proponents argue that this model is most suitable to safeguard individuals against value‐based judgments of healthcare professionals about whether their lives are still worth living when it comes to decisions on assisted dying. In this paper, I challenge the assumption that the autonomy‐only approach successfully avoids the shortcomings of a joint view at assisted dying that also relies on beneficence‐based criteria. Based on the example of Germany, a country that follows the autonomy‐only approach towards assisted suicide, I contend that, despite its emphasis on personal freedom, this approach may in practice not truly serve the ideal of autonomy as it claims. Referring to critical medical ethics, I argue that Critical Theory offers the epistemic tools needed to identify underlying social contradictions and to evaluate the concept of autonomy in relation to its real‐world application. Drawing on the work of Herbert Marcuse, I highlight the importance of considering how individual needs are shaped and mediated by social conditions in ethical deliberations on assisted dying. I conclude that ethical considerations have to avoid both submitting to short‐sighted conceptions of autonomy and taking a paternalistic stance that dismisses individual pledges for assisted suicide if they disagree with supposedly objective reasons.

## 1 Introduction

In 2020 the German Federal Constitutional Court dealt with assisted suicide services—not voluntary euthanasia—and decriminalized them by court decision.^1^ Since then, voluntarily given assistance with suicide is increasingly practiced in Germany and in most cases carried out by right‐to‐die organizations [1]. The court considers a self‐determined death part of the general right of personality, thus justifying it with a fundamental right that has to be protected as part of human dignity [2]. Furthermore, the German situation is unique as there is—as yet—no law which further regulates access to assisted suicide (AS). Although the legislator can further regulate access by law, the court has set limitations to exclusively safeguard the individual will, stating that the right to a self‐determined death is not limited to a certain age, a medical condition or a state of unbearable physical or mental suffering.^2^

It is quite unusual for the decriminalization of any form of assisted dying by court decision that its justification only relies on the individual will [3]. In the ethical debate this has been discussed as an autonomy‐only approach that does not refer to beneficence‐based criteria, which usually require that someone experiences suffering which is unbearable to justify assisted dying [4, 5, 6]. Proponents claim that an autonomy‐only approach is able to avoid the indirect paternalism of a joint view that to some extent objectifies an ultimately subjective experience, namely suffering, usually by tying it to a medical condition [4, 7]. In contrast, recent articles have criticized the concept of autonomy that the court decision in Germany refers to, especially when considering the vulnerability of suicidal people and people in end‐of‐life situations [8, 9, 10]. While I agree with the criticism of the joint view, I will argue that

1. An autonomy‐only approach may not be able to avoid the shortcomings of the joint view as claimed by its proponents.
2. At the same time, there is in danger of neglecting ethically often demanded protective measures.
3. The court decision in Germany pre‐sets a *certain* understanding of individual will that poses a challenge for some self‐conceptions of medical ethics.

Thus based, one could argue that the current situation in Germany is more liberal than what might be ethically demanded, especially when regarding the absence of public policies or laws and missing evidence on the usage of AS under the new legal situation [11, pp. 185–186]. However, these challenges will not make me pledge to stricter regulations. Rather, I argue that we should ask very carefully whether current practices of assisted suicide are actually suited to give expression to the concept of autonomy they refer to—in the interest of maintaining the agency of people willing to die. To illustrate this, I am drawing on the approach of critical medical ethics [12]. To expand this perspective on AS, I will furthermore refer to Herbert Marcuse's idea of a sensible cultural arrangement of dying. Using this approach, I argue that there is a discrepancy between the claim of autonomy and the actual AS practice in Germany. I conclude that ethical considerations have to avoid both submitting to a problematic conception of autonomy *and* taking a paternalistic stance that dismisses individual pledges if they disagree with supposedly objective reasons [13].^3^

## 2 Assisted Dying and the Problems of the Joint View

Most legislations of assisted dying refer to autonomy and beneficence‐based criteria (joint view) [6]. They usually require suffering that is unbearable, cannot be relieved otherwise and stems from a medical condition. This criterion can justify assisted dying to be in somebody's best interest, thus being an act of beneficence. In the last years, the joint view has been increasingly criticized in the ethical debate, sometimes in combination with referencing to the German court decision as an example for an autonomy‐only approach [4, 6, 7]. Such an approach holds that AS is morally permissible when it is based on an autonomous request that can be made also for reasons other than medical ones, which means that not necessarily it needs to be understood as a medical procedure [4]. This allows for a broader range of cases to be acceptable for AS, especially regarding people being tired of life without any diagnosable medical condition [6]. Critics argue that assisted dying laws that require beneficence‐based criteria express the judgment that certain stages of illness are not worth living and are thus discriminating. In addition, they can be a gateway for indirect paternalism based on value judgments of others [4, 7]: Even though the category of suffering is an inherently subjective one, it is to some extent objectified and submitted to external evaluation, usually by tying it to the existence of a medical condition. Thus, physicians can refuse assistance under the argument that it is not in a patient's best interest because they differ in the assessment of the patients' medical situation or whether their lives are still considered worth living. According to proponents of an autonomy‐only approach, solely justifying access to AS with the autonomous request of a person whose reasons should not be judged on by others is better suited to avoid these problems.

I agree with these arguments regarding the problems of a joint view as well as with not understanding AS as a medical procedure. However, while I agree that an autonomy‐only approach might be less susceptible for indirect paternalism and value judgments, by the example of Germany I would like to challenge the assumption that an autonomy‐only approach will be able to avoid these deficiencies. The autonomy‐only judgment in Germany aims to protect the autonomous will against value judgments of others by saying that state and society have to respect the individual assessment of a meaningful life by referring to fundamental rights, namely the freedom of opinion and the freedom to unfold one's own personality [2]. Access is regulated only via the ability to form an autonomous and free will *(Freiverantwortlichkeit)*, which includes the following criteria: having decision‐making capacity, which is understood accordingly to informed consent, the absence of undue influence or pressure, and a degree of internal stability [2].

However, for an autonomy‐only approach to succeed we have to put into question whether there can be an ‘objective’ assessment of an autonomous decision that avoids the above‐mentioned shortcomings while at the same time still depending on the professional assessment of someone else. Considering this, medical professionals and relatives have to be able to differentiate between the subjective reasons of a patient, that are not necessarily comprehensible to be legitimate, and his or her competence. But, as Beauchamp and Childress point out to, different value conceptions about what is in the best interest of patients also at times find expression by questioning their decision‐making capacity [14, p. 141]. There are indications that, with growing emphasis on individual decision‐making regarding one's own care planning, people whose decision‐making capacity is in question are specifically endangered to be exposed to paternalistic practices, for example in the context of dementia: One study in Germany pointed out that the involvement of family members or healthcare professionals in the preparation of an advance directive is not seen as something that needs *any* justification or explanation—even if the person had not even been diagnosed with dementia at the time the advance directive was prepared [15].

As of now, I cannot indicate the magnitude of the problem that different value conceptions are reason for questioning decision‐making capacity regarding AS in Germany, as there is very little evidence on current assessment procedures. However, there has been a court case in Spain which is particularly representative of this problem: There, a father suspended the euthanasia process of his adult paraplegic daughter in court with the help of the campaign group Christian Lawyers after a multi‐professional evaluation board with access to her medical reports had unanimously supported her decision [16].^4^ Interestingly, the lawyers primarily argued that a personality disorder affected her judgement (thereby questioning her autonomy) and only on that base claimed that granting AS is an act of maleficence in such cases (thereby questioning beneficence). The woman, however, reaffirmed her will to die and stated that she had felt coerced by the religious groups that supported her father in challenging her decision.

One can now argue that if the decision‐making capacity is in doubt in similar constellations, people can just refer to a different physician or organization for a new assessment. However, there are indications that after having been denied assisted dying, people usually do not ask for it again somewhere else, although their wish to die does not necessarily subside [7, 17].^5^ Based on the observations I have presented, I argue that an autonomy‐only approach does not per se solve this problem, as people's autonomy can also be overridden by questioning their decision‐making capacity. In fact, fighting a wrong assessment of capacity might even be more difficult than fighting a wrong assessment of the suffering criterion, as it is generally assumed that the former is more objective. In such a situation, we should elaborate very carefully on the scope of human interpretation that an autonomy‐only approach leaves us with – and how we handle its other challenges.

## 3 Assisted Dying and the Protection of Autonomy

Regarding end‐of‐life decision‐making, several studies point out to the complexity of assessing patients' death wishes, showing that these are often incoherent and can change over time [18, 19]. The changing nature of a death wish does not mean that requests for assisted dying cannot be autonomous in general. However, if we agree that suffering is an ultimately subjective category, an approach that solely relies on autonomy particularly demands asking how to adequately protect the autonomy of people whose will to die might result from a vulnerable situation that is not assessable for others.

One could argue that this can be overcome by establishing legal regulations combined with evaluation processes. This is the task of empirical ethics: to constantly assess the frequency of the usage of AS and to analyze sociodemographic factors, to see whether certain vulnerable groups are specifically susceptible to reaching back to AS [20]. Thus based, Beauchamp and Childress propose a precautionary principle regarding the rules of practice, especially as long as there is little evidence of the development of practices of assisted dying in a certain legal situation [11, pp. 185–187]. They argue that laws to protect individuals against abuse can be appropriate, while at the same time individual actions that defy them are not reprehensible. However, this only reflects upon a situation in which law renders some acts of AS illegal that might be ethically justified. But in Germany the situation currently presents itself the other way round, with public policies yet waiting to be established, which constitutes a problem for the protection of autonomy. As first and rather preliminary research indicates, current AS practices in Germany tend to neglect measures that are often called for in the ethical debate on protecting autonomy in such vulnerable situations: Oftentimes there was no ongoing physician‐patient relationship, no separation between the person assessing the free will and the person assisting with suicide, it was not traceable whether alternatives to suicide were indicated, and the costs for AS were usually in the high four‐figure range, which can constitute both pressure and an accessibility problem [1, 21, 22].

Furthermore, two court decisions in Germany prosecuting physicians who had performed AS on patients with mental illnesses have undermined the possible challenges of an autonomy‐only approach in combination with lacking legal regulation [23, 24]. One of these cases particularly points out to the difficulty of proving a clear consistency of will in the context of depression. In legal debates, this case has foremost been referred to as illustrating the legal risks for physicians to provide AS in cases of mental illness [25]. In my opinion, it primarily raised several questions of ethical relevance regarding the procedures which precede AS: how to handle unsuccessful attempts, whether the physician assessing the death wish should have to be specialized in the underlying medical condition if access ultimately does not depend on the state of the condition, and how to handle doubts expressed by the person before their death. Regardless of whether it was ultimately right or wrong that the person died as a result of AS, the case is thus in many aspects systematic for the challenges an autonomy‐only approach in combination with missing legal regulation can bring along.

## 4 A Certain Understanding of Individual Will

The third point I would like to assess is a certain understanding that the court decision expresses when stating that the will of the individual “eludes any appraisal on the basis of general values, religious precepts, social norms for dealing with life and death, or considerations of objective rationality” [2, para. 210]. As I have already indicated, arguing that the state—or any other person or institution—should not judge on individual reasons shows a progressive element to protect people against value judgments of others. However, accepting the prioritization of the individual will above objective rationality poses a challenge for certain disciplinary self‐conceptions of ethics. While it is common that ethical considerations are not the final decisive factor when it comes to medical decision‐making (see the advisory function of ethical case discussions), they are evaluative statements based on reasonable arguments while considering different perspectives. A principlism‐based approach itself is an example of such reasoning, as it derives its *prima facie* principles from a supposed “common morality”, and Beauchamp and Childress “appropriately judge all human conduct by its standards” [11, p. 3]. However, the German judgement not only says that such considerations cannot be decisive but that the will of the individual eludes being assessed this way, which constitutes a very absolute understanding of autonomy [10]. But when thinking about how inherently the shaping of peoples' values and their understandings of concepts such as autonomy are embedded in social processes, the aspired separation between individual will and any way to assess its formation relating to general values or rationality constitutes an explanatory gap between ethical theory and practice: As a systematic integrative review has found out, empirical bioethical studies hardly ever discuss the link between individual understandings of autonomy and social possibilities of its realization but rather already presuppose autonomy as a central value behind assisted dying [26].

To further evaluate the consequences that might arise from an autonomy‐only approach to AS, we can, in my opinion, not only rely on empirical ethics to point out the consequences of socio‐ethical developments and call on stricter regulations based on levels of evidence. Following this path, we run the danger of missing linking scholarly conceptions of autonomy to the social and cultural embedding of personal understandings of autonomy as well as of neglecting the ambivalent nature of pre‐suicidal reasoning [27]. In addition, we stay within an expert debate about how certain normative principles we already preset should or should not be embedded in law [12]. Rather, I propose to draw on an approach that reflects on the intrinsic connection between individual and socio‐ethical aspects not only by collecting evidence but also in the way it refers to epistemic categories such as autonomy. In the following, I will base this argument on the approach of critical medical ethics.

## 5 The Approach of Critical Medical Ethics

In his article *Foundations of Critical Medical Ethics* Giovanni Rubeis states that medical ethics as an academic discipline is constantly confronted with transforming social terms of action for medicine, as changing economic and technological developments produce new individual and collective challenges [12]. He argues that medical ethics cannot meet these challenges by relying solely on new empirical facts and studies, as suggested by the so‐called empirical turn. According to Rubeis, this is not only a problem of changing the medical practice but of changing the epistemic categories according to which we analyze social reality and power asymmetries. Rubeis proposes that the thought school of Critical Theory of the Frankfurt School can contribute to reflecting on the epistemic categories to capture and classify the general conditions under which physicians and patients act and make moral decisions. There is as yet, at least to my knowledge, no systematic reception of Critical Theory in the international discourse of medical ethics. Accordingly, the approach of critical medical ethics is rather preliminary and further elaboration is needed. Regarding end‐of‐life situations, the concept of relational autonomy, which grasps intersubjective dimensions of decision‐making procedures but also conceptions of feminist bioethics that focus on vulnerabilities and power asymmetries, might further contribute to this project [12, 28, 29].

In brief, Critical Theory combines elements of philosophy, social science and psychoanalysis to formulate a critique of society with a claim for radical social change and human emancipation from heteronomous conditions [30, 31]. Thereby, it integrates a normative perspective with an “empirically informed analysis of society's conflicts, contradictions, and tendencies” [32].^6^ For the purpose of this article, I intend to focus on one concrete idea of Critical Theory and its potential for a debate about AS: the idea that, by comparing concepts such as autonomy with their subject matter, Critical Theory can provide us with the epistemic categories to recognize social contradictions and eventually grasp actual possibilities to act [12]. To illustrate this, I will reference one of Critical Theory's most prominent representatives, Herbert Marcuse. Marcuse's thoughts are specifically intriguing in the way they pinpoint social contradictions and critically question the categories with which common rationality operates. At the same time, his thoughts enable to understand why it is so hard to see through these contradictions that became a social reality and thus why, according to Critical Theory, a rational organization of society according to human interests is so difficult [33]. If so, it is defined very vaguely as the “rational organization of the realm of necessity”, that is the space where material needs are satisfied, to obtain a “free development of needs on the basis of satisfaction” [34, p. 239]. But what does obstruct this sort of organization in current social conditions?

To better understand this, Rubeis pointed towards the category of totality as one that can fuel the debate about critical medical ethics: Totality does not refer to any totalitarian political organization of society but to “a non‐terroristic economic‐technical coordination which operates through the manipulation of needs by vested interests” [34, p. 5]. It thus articulates that social and cultural institutions constitute some sort of totality towards individuals, as they did not autonomously shape them, yet they appear to them as a closed ‘total’ formation. Social determinants such as power asymmetries shape the ways in which individuals can exercise autonomy. The concept of autonomy thus often refers to the capacity to decide within a certain acceptable framework of reasoning.

In liberal constitutional states, the legal system plays a decisive role in defining “what can be chosen and what is chosen by the individual” [34, pp. 9–10], as it provides the framework within which it is possible for individuals to experience themselves as being autonomous [35]. For example, Graefe argues that the judgment in Germany sets the granting of AS as a guarantor of individual freedom and thereby already places this idea above the protection of life [36]. As she argues, this reduces personal freedom to choosing between different options while at the same time exaggerating it as an absolute freedom that transcends even the *de facto* lack of freedom. From a critical perspective, it is therefore necessary to look at the socioeconomic developments that increasingly bring the shaping of one's own death to individuals as a requirement.

Another important aspect of Marcuse's thinking is his extensive critique of technological rationality according to which modern societies are organized. One example of the twofold nature of such rationality are technological achievements in medicine, which have brought forward better care possibilities and a higher life expectancy. In Marcuse's terms, they have thus contributed to a better fulfilment of human needs and even conquest nature to a certain extent [37, pp. 3–4]. At the same time, the very same developments also require individuals to come to terms with which states of independence and dependence—on other people or on technological aids—they still consider worth living in, especially with increasing age [38]. They have, on the one hand, created the freedom for individuals to decide about such situations [39].^7^ On the other hand, they also *demand* those decisions from us [40]:^8^ As social security systems have increasingly individualized risk management in the last 20 to 30 years, there is, ideally, no decision that should be left open when it comes to shaping our lives and deaths authentically in accordance with our own values and self‐perceptions [35, 41]. As a result, deciding autonomously becomes a possibility but also a requirement with regard to the organization of one's own death.

These two motives are meant to illustrate that social institutions and structures, including the legal system, not only deeply influence the wish for individual self‐determination. They also produce the conditions in which self‐determination can be realized to such an extent that we have to reject the separation between individual and socio‐ethical aspects in debates about assisted dying that is usually conceptualized in medical ethics. But Critical Theory does not stop there—it derives a transformative claim from such analysis: the need to properly articulate how individuals refer to such concepts as part of a social process that in many aspects is out of the individuals' control points out the necessity to change these conditions. As Rubeis argues in reference to Herbert Marcuse, Critical Theory does so by examining to which extent concepts are appropriate to their subject matter [12]. Regarding a self‐determined death, this means that we not only need empirical bioethics but also social‐theoretical and epistemic reflections on what the concept encompasses and whether the possibilities to shape one's own death can *actually* address and express these aspirations. But how can Critical Theory help us grasp the concept of a self‐determined death?

## 6 A Sensible Cultural Arrangement of Dying

In one of his most influential works, *Eros and Civilization*, Marcuse examines the relationship between individual freedom and cultural progress based on a sociological reading of Sigmund Freud. According to Freud, civilization is based on the subjugation of human instincts and their mediation in a socially acceptable way. Marcuse challenges the assumption that the “suffering thereby inflicted on individuals” [37, p. 3] is as inevitable as Freud claims, by stating that it is rather the result of a “specific historical organization of human existence” [37, p. 5]. Despite his negative diagnosis of a repressive civilization, Marcuse's thoughts transport a positive idea of what a sensible arrangement of dying might look like that I consider relevant for dealing with death in modern culture:

Death can become a token of freedom. The necessity of death does not refute the possibility of final liberation. Like the other necessities, it can be made rational—painless. Men can die without anxiety if they know that what they love is protected from misery and oblivion. After a fulfilled life, they may take it upon themselves to die—at a moment of their own choosing. But even the ultimate advent of freedom cannot redeem those who died in pain. It is the remembrance of them, and the accumulated guilt of mankind against its victims, that darken the prospect of a civilization without repression.

[37, pp. 236–237]

At a first glance, invoking this citation as an ideal, one might rightfully argue, contradicts the rejection of beneficence‐based criteria I have supported above, as it refers to pain and anxiety. However, with Marcuse's approach, we can try to understand suffering not as an objectifiable criterion but as a social phenomenon and thus as a shared form of experience that constitutes empathy. According to him, the ultimate objective of human instinct is the termination of pain, which is not possible in a culture that has perpetuated suffering [37]. He not only refers to physical pain here but also to the anxiety that what we love may suffer “from misery and oblivion.” According to him, the prospect of death being inevitable not only drives on medical progress such as the technological achievements I have mentioned above but also cultural mechanisms of repression, as we all learn to how accept death as an inevitable necessity from very early on. One expression of such repressive cultural mechanisms is the cold indifference towards conditions of existence under which for many it seems to make no appreciable difference whether a person dies “naturally” after a fulfilled life or earlier and in more suffering than she would perhaps have wanted under other conditions—at least not a difference that is worth fighting for with all instinctual energy. That is how, according to Marcuse, in a repressive civilization “death itself becomes an instrument of oppression” [37, p. 236].

He justifies this with the historic guilt a civilization has toward people who died in unnecessary pain, which gives mankind the responsibility to create conditions under which no one has to die earlier or in more—emotional or physical—pain than necessary. Technically, the resources to change this are available—but the distribution and production of modern society does not allow for their usage in the service of meeting all basic human interests. As death is an inevitable part of human existence and as he identifies the desire to die without fear and pain as being deeply human, one minimal criterion would thus be to make it painless and fearless for everybody. This way, Marcuse's approach consequently exemplifies how, by working through social and cultural contradiction, we can state—if only to the slightest extent —an idea of what could be done differently. Thus, it should drive forward our aspirations to find a sensible cultural arrangement of dying.

For him, another expression of such repressive mechanisms is the silent “professional agreement” towards the fact of death and disease [37, p. 236]. This is not to say that it is the fault of physicians if people resign when facing illness or pain—that would be presumptuous, especially after all the remarks about the social embedding of death wishes. It rather means that the physician assessing whether we meet the criteria for a self‐determined death has power over the interpretation and social acceptability of a death wish, be it conscious or unconscious. In an ethnographic study on suicidality in psychiatry, Iltzsche observed a process that I consider representative in this respect: He describes how the treating psychiatrist legitimized an elderly person's wish to die by no longer understanding it as an expression of pathological suicidal tendencies but by rather reinterpreting it as being rational in the context of a certain physical condition [42]. This framework allowed the psychiatry to accompany the patient with voluntarily stopping eating and drinking. Iltzsche concludes that the patient was ascribed the agency that legitimized his wish to die by a psychiatrist who put herself in an all‐knowing position.

Invoking this example showcases that the assessment of a death wish being dependent on an ascribed medical expertise inherently carries a heteronomous element [39, 42]. That would both allow for a medical paternalism that de‐legitimizes the death wish by questioning the patients' decision‐making competence as well as for physicians rationalizing a wish to die in the context of their own interpretation. It also illustrates how dependence on others is inherently embedded in assessment procedures, thereby questioning whether an absolute conception of autonomy can do justice to the inherently social nature of this process [10]. Here, it is also worth looking at the extensive measures right‐to‐die organizations in Germany take to ensure the free will of those they assist with suicide, as this is crucial for legal AS [1, 21]. Their documentation processes include an advance declaration of the suicide wish, a release from the physicians' obligations as a guarantor, an assessment of free responsibility by a physician, a declaration of suicide, and a detailed protocol of the dying process. In addition, at least one doctor and often a lawyer are present at the time of death, and sometimes the suicide is recorded to later provide evidence. I do not want to contend that the involvement of other people and some form of documentation is necessary surrounding AS procedures. I mainly want to point out that the professionalization and rationalization of death comes at the price of autonomy being mainly reduced to a formalized procedure of ensuring informed consent and ultimately opening a turning device.

However, it is very important to note that I do not intend to de‐legitimize individual requests for AS based on my critique. As I have been trying to make comprehensible by the concept of totality, social conditions enforce themselves through individuals, even though at the same time they reproduce them by way of their actions. We can further try to understand this when looking at other motives and meanings of wishes to die. While the current debate about assisted suicide predominantly circles around the principle of autonomy, people who are actually interested in hastening death rather speak of the fear of losing physical or internal control than of realizing their autonomy [26]. A lack of prospects in life, the perception of one's own life as being worthless, tiredness of life or fear of needing care were named more frequently as the main motive for wanting to die [43]. Other related expressions include the fear of loneliness and fear of a loss of dignity. In addition, the unwillingness to depend on others and the subjective perception of being a burden were important concerns for patients in advanced care situations [44, 45]. Here, the connection with social conditions is especially evident when looking at the commercialization of medicine. I understand commercialization as a process in the course of which the delivery of healthcare services is increasingly formed by calculations of profit or advantage [46, 47]. Then the social relation between caregiver and caretaker to a certain extent becomes a means to the end of profitable treatment. In that regard, it is understandable that people are afraid of being dependent on others and cannot perceive this dependency as a neutral or even positive and strong social relation. In fact, we can hardly expect individuals to feel different when being confronted with the prospect of becoming old in an overburdened care system.

The decisive question now is: What can we say about the legitimacy of individual will if we reject the possibility that autonomy can be realized to the extent an autonomy‐only approach requires? The point I am trying to make is not about presenting a fundamental argument pro or against AS. Rather, I want to contribute to an understanding of requests for AS in a society where AS is a social reality, while at the same time the conditions for realizing autonomy remain precarious. Even while the individual decision may be the result of a conscious thought process in regard of one's own situation, it is oftentimes not made free of anxiety or suffering caused by social circumstances. According to Marcuse, this is reason enough to change our cultural arrangement of dying with all our energy. At the same time, it should also be reason enough to strengthen peoples' agency as best as possible—even if ultimately this means assisting them with suicide as the last act they *could possibly* perceive as autonomous. In that regard, the “right” point of dying from a medical standpoint might not be the right one for the individual: what is, medically spoken, earlier than necessary can, individually spoken, be a situation someone did not want to experience and thus already “too late”. This is the neuralgic point in the discussion of autonomy and AS: by its idea, an autonomy‐approach forbids to expect people to continue living based on a value judgment of their situation by someone else, as this would also be a way to limit their choice. This way, putting individual will above objective rationality gives individuals the powerful right to make even “irrational” choices. In a society that has a problematic understanding of rationality itself, this can be a relief and, arguably, one of the few refuges to *resist being rationalized by others*. This said, we would have to add to Marcuse that of course no one should have to die earlier than would have been necessary and possible (or *feel* like they have to)—but also not later than they *want to* just because it would have been within the medical realm of possibility. However, we could only be sure about the authenticity of individual will regarding the time and way of dying if, in the Marcusean way, we liberated death from heteronomous circumstances, if there *was* a possibility do die without all social anxiety. Until then, we will be left with the social exercise to deliberate how much insecurity we are willing to accept under an autonomy‐only approach in a situation when the chances of realizing autonomy are precarious.

In reference to Rubeis and Marcuse, I have argued that questioning whether a social order is actually equipped to express the human interests it refers to is an inherently ethical question, as it determines whether epistemic concepts are appropriate to describe the social reality they apply to. As I have pointed out by the example of Germany, the current practice does not succeed in that regard, as it actually shows tendencies that ethically often required measures to protect autonomy tend to being neglected, while the assessment of an autonomous will tends to being an extensively formalized procedure. This constitutes a problem with two possible tipping points: First, if we rely too much on regulations and the supposed objectivity of professional assessment, we risk falling into paternalism and the value judgments of medical professionals that are hard to conquer. Second, if we only provide the framework of an autonomy‐only approach without critically reflecting on the social reality it applies to, we risk missing a transformative claim: When asking how the moral content of autonomy can be realized in current social practices, we would have to add with Marcuse that at first we have to create conditions under which people *could* choose differently than is currently the case. This is the most important condition for developing a sensible cultural arrangement of dying that is oriented at human interests— some of whom people might not yet be able to give expression to. It challenges us to think about the unconditional simultaneity of assisted suicide and a necessary change of the conditions that give rise to the need for assisted suicide in the first place.

Against this background, I pledge to shift the focus of the ethical debate to transform AS into a social practice that represents the desired autonomy. In this debate, medical ethics should not limit its contributions to deliberating and evaluating procedural safeguards or assessment criteria but critically point out the social function of legal regulations and medical expertise themselves. As I have demonstrated, when justifying AS solely autonomy‐based, this especially entails the social responsibility to reflect on why certain options appear more or less acceptable to people than others. In addition, it demands to have a look at what *can actually* be chosen by people and how autonomy is assessed and exercised in procedures of AS.

## 7 Conclusion

By the example of Germany, I have argued that an autonomy‐only approach cannot avoid the problems of a joint view as well as its proponents claim while at the same time it runs the danger of neglecting ethically often demanded protective measures. Furthermore, I have shown that the court decision in Germany pre‐sets a certain understanding of individual will, which poses a challenge for certain disciplinary self‐conceptions of ethics. Here, medical ethics should not limit its contribution to collecting evidence but rather reflect on the intrinsic connection between individual and socio‐ethical aspects and the way epistemic categories can refer to this in ethical debates. By the approach of critical medical ethics that I have pledged for, we can examine how the concept of a self‐determined death finds expression by the will of individuals, by the current legal system and by what we expect from physicians in this context. As I have argued with Herbert Marcuse, this not only entails questioning whether the concept of autonomy can actually be realized in current social practice but also strengthening the possibilities of individuals *to be able to decide differently*. This includes liberating individual will from as much social anxiety, suffering and prefabricated decision‐making as possible. I concluded that a critical ethical perspective can help protect requests for AS against both paternalist definitions by others and against an indifference to circumstances that may bring about this desire earlier or with more suffering than necessary.

## Data Availability

The author has nothing to report.

## Endnotes

1 The term assisted suicide describes a process in the course of which the person willing to die ultimately performs the act that leads to death but is provided with the means to do so (like lethal medication) by someone else. In cases of voluntary euthanasia, the act that leads to death is carried out by somebody else on request of the person willing to die, usually a medical professional. Assisted dying is a broader term encompassing both assisted suicide and voluntary euthanasia. In some countries, like the Netherlands, both forms are permissible and the distinction is not legally relevant. In Germany, however, voluntary euthanasia remains illegal as ‘killing on request’. Thus, when referring to Germany I will exclusively refer to assisted suicide while sometimes using the broader term when reflecting on the general ethical debate about assisted dying.

2 Technically, the conditions in Switzerland are similar: access is not regulated by law, and only assisted suicide for selfish motives is punishable. However, in Switzerland there is a long‐established practice of right‐to‐die organizations that usually demand irremediable suffering as a criterion for access to assisted suicide. In Austria, at the end of 2020 the decriminalization of AS was effectuated by a court decision with similar reasoning. However, in 2022 a law was introduced there that describes procedures in detail. The formal requirements include suffering from terminal or serious, permanent illness.

3 This pledge is in accordance with a critique on the current debate to be dominated by a liberal individualism on the one side and a sceptic professionalism on the other as it was sketched in a recent anthology that tried to bring forward a critical debate in Germany. See Duncker and Schmidt [13].

4 There are no scientific articles published on this yet. However, cases like these receive increasing attention in public and academic discussion as I have learned a recent conference on end‐of‐life issues in Spain.

5 Holzman has pointed to this as a problem of moral relevance of the Dutch approach, but only because of the physicians' deciding power regarding the suffering criterion.

6 However, the normative reading of Critical Theory, especially of their older representatives, is disputed.

7 Simon Duncker has pointed out this twofolded character of Foucault's theorem of biopolitics.

8 These developments have been discussed in medical ethics in reference to the theorem of biopolitics by Michel Foucault.
